# Fabrication and Characterization of CMOS-MEMS Magnetic Microsensors

**DOI:** 10.3390/s131114728

**Published:** 2013-10-29

**Authors:** Chen-Hsuan Hsieh, Ching-Liang Dai, Ming-Zhi Yang

**Affiliations:** Department of Mechanical Engineering, National Chung Hsing University, Taichung 402, Taiwan; E-Mails: bad_fox_2003@hotmail.com (C.-H.H.); d099061005@mail.nchu.edu.tw (M.-Z.Y.)

**Keywords:** magnetic sensor, Lorentz force, CMOS, post-process

## Abstract

This study investigates the design and fabrication of magnetic microsensors using the commercial 0.35 μm complementary metal oxide semiconductor (CMOS) process. The magnetic sensor is composed of springs and interdigitated electrodes, and it is actuated by the Lorentz force. The finite element method (FEM) software CoventorWare is adopted to simulate the displacement and capacitance of the magnetic sensor. A post-CMOS process is utilized to release the suspended structure. The post-process uses an anisotropic dry etching to etch the silicon dioxide layer and an isotropic dry etching to remove the silicon substrate. When a magnetic field is applied to the magnetic sensor, it generates a change in capacitance. A sensing circuit is employed to convert the capacitance variation of the sensor into the output voltage. The experimental results show that the output voltage of the magnetic microsensor varies from 0.05 to 1.94 V in the magnetic field range of 5–200 mT.

## 1. Introduction

Magnetic sensors are important devices for diverse applications in industrial equipment and electronic instruments. Microsensors have the advantages of small size, high performance, low cost and easy mass-production [1]. Several traditional magnetic sensors were miniaturized as magnetic microsensors using microelectromechanical system (MEMS) technology. For instance, Yin *et al.* [2] used MEMS technology to fabricate a microcantilever torque magnetic field sensor. The sensor consisted of a torsion oscillator and a soft magnetic Fe_77.5_Si_7.5_B_15_ wire, and it had an ability to detect magnetic field changes of a few nT under ambient conditions. Du *et al.* [3] proposed a piezoresistive magnetic field sensor with a silicon bridge structure manufactured by MEMS technology. The sensor contained a silicon diaphragm embedded with a piezoresistive Wheatstone bridge, and a ferromagnetic magnet adhered to the diaphragm. The magnetic force bended the silicon diaphragm when subjecting to an external magnetic field to the sensor, and the resistors of the Wheatstone bridge changed. The sensitivity of the sensor was 48 mV/T. A micro fluxgate sensor with solenoid coils, presented by Lei *et al.* [4], was also fabricated using MEMS technology. Solenoid coils were adopted as excitation and sensing elements in the fluxgate sensor. The magnetic core material was an iron-based nanocrystalline alloy. The greatest sensitivity of the sensor was 583.1 V/T with the effective excitation current of 150 mA. Brugger and Paul [5] employed MEMS technology to develop a resonant magnetic microsensor with a shift of the mechanical resonance frequency. The sensor was composed of an electrostatically driven micromechanical resonator with a planar magnetic concentrator with two narrow gaps. The sensitivity of the sensor was 1.91 MHz/T with a coil current of ±120 mA. Marauska *et al.* [6] developed a MEMS magnetic sensor based on magnetoelectric composites with vacuum encapsulation using wafer-level packaging technology. The magnetic sensor was made using micromachining process and bonded afterwards for hermetic sealing. The sensor included rectangular cantilever beams with a stack consisted of SiO_2_/Pt/AlN/FeCoSiB, Au metal-bond frames and conduction lines. The sensitivity of the sensor was 3,800 V/T at the resonance frequency of 7.1 kHz. Choi *et al.* [7] developed a torsional resonant magnetic sensor combined with a permanent magnet supported by multiple micromachined silicon beams. The magnetic sensor had a low power consumption of 140 μW and a sensitivity of 0.28 Hz/rotational degree. The area of the sensor was 5 × 5 mm^2^. Li *et al.* [8] used MEMS technology to fabricate a three-axis Lorentz-force magnetic sensor. The device was a micromechanical resonator, and the sensing was conducted using excitation currents at the resonant frequencies of the device's in-plane and out-of-plane. The sensor had a power consumption of 0.58 mW and an offset of 400 nT with a 0.7 s. The area of the sensor was 1 × 0.2 mm^2^. Langfelder *et al.* [9] employed an industrial MEMS technology to manufacture a z-axis magnetometer for MEMS inertial measurement unit application. The sensitivity of the sensor was 150 μV/μT at the driving current of 250 μA. Dominguez-Nicolas *et al.* [10] developed a small magnetometer based on MEMS technology for detecting respiratory magnetogram. The sensor had a resolution of 20 nT. Thompson and Horsley [11] employed MEMS technology to develop a Lorentz force navigation magnetometer. The device had a noise resolution of 87 nT/√Hz and a corresponding angular resolution of 0.7 °/√Hz. Tapia *et al.* [12] presented a resonant magnetic field sensor fabricated using MEMS technology for detecting the spiking activity of neurons and muscle cells. The magnetic sensor was composed of an array of silicon beams and p-type piezoresistive sensing elements. The sensor had a low power consumption of 2.05 mW, a resolution of 80 nT and a sensitivity of 1.2 V/T. Wu *et al.* [13] proposed a magnetic field sensor consisted of a square extensional mode resonator with a planar induction coil. The sensor utilized capacitive driving and electromagnetic induction sensing method to detect the external magnetic field. The sensitivity of the sensor was 3 μV/mT. The magnetic sensor needed a vacuum packaging to enhance its performance. Their sensors [2–11] were not integrated with circuitry. In this work, we develop a magnetic sensor integrated with a sensing circuit.

Several microdevices have been fabricated using the commercial CMOS process [14–17]. Micro- devices manufactured by this process have the potential for integration with circuitry on-a-chip [18–21]. In this work, we developed a magnetic microsensor using the commercial CMOS process. The magnetic sensor needs a post-process to release the suspended structure. The post-process includes an anisotropic dry etching to remove the sacrificial oxide layer, an isotropic dry etching to etch the silicon substrate and a wet etching to etch the aluminum layer. When the magnetic sensor detects a magnetic field, the capacitance of the sensor generates a change. A sensing circuit converts the sensor capacitance into the output voltage.

## 2. Structure of the Magnetic Sensor

**Figure 1.**
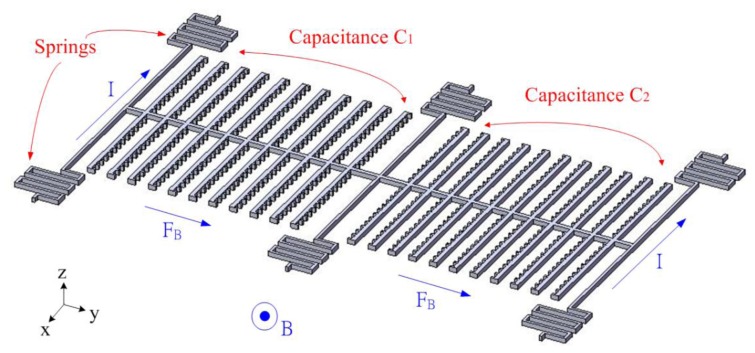
Schematic structure of the magnetic sensor, where *I* is current; *B* is magnet; *F_B_* is Lorentz force.

Figure 1 illustrates the schematic structure of the magnetic sensor. The magnetic sensor contains six springs and two set of interdigitated electrodes. The springs are used to support the interdigitated electrodes. As shown in Figure 1, two set of interdigitated electrodes form a differential capacitance couple, where one is capacitance *C_1_* and the other is capacitance *C_2_*. The magnetic sensor is actuated by the Lorentz force, and this force is induced by magnetic field. The Lorentz force is given by [22]:

(1) $$F_{B}=IL\times B$$

where *F_B_* represents the Lorentz force; *I* is the current; *L* is the length of conductor and *B* is the magnetic field. According to Equation (1), we know that the Lorentz force depends on the current and magnetic field.

**Figure 2.**
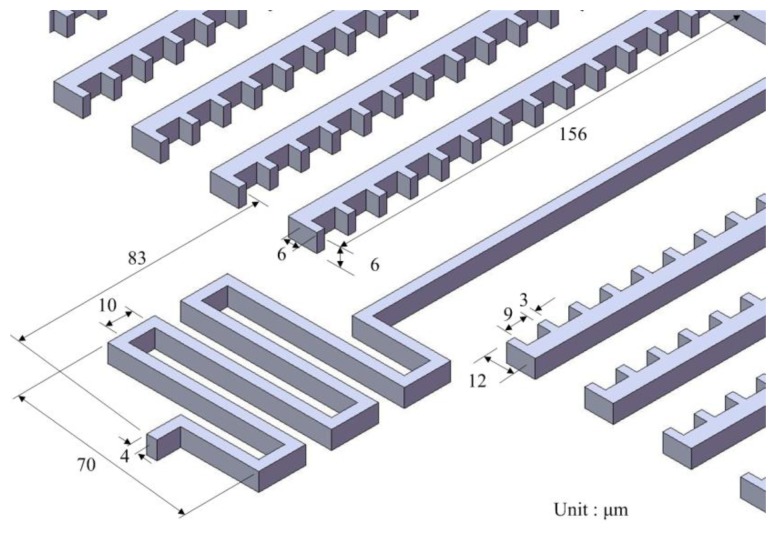
Dimensions of the magnetic sensor.

As shown in Figure 1, when supplying a current to the springs in the −x direction and applying a magnetic field in the z direction, the Lorentz force is induced in the y direction. The Lorentz force drives the two set of interdigitated electrodes causing a displacement, and making the capacitances *C_1_* and *C_2_* change. The capacitances *C_1_* and *C_2_* are designed as a pair of differential capacitances. The capacitance *C_1_* undergoes a capacitance increase of *ΔC* as the capacitance *C_2_* undergoes a capacitance reduction of *ΔC*. The advantages of a differential capacitance include an enhanced common-mode rejection ratio (CMRR) and reduced harmonic noise. A sensing circuit is utilized to convert the capacitance variation into the output voltage. Figure 2 shows the dimensions of the springs and interdigitated electrodes. The number of interdigitated electrodes is 22, and the number of fingers in each electrode is 13. The gap between the fingers of electrodes is 3 μm.

**Figure 3.**
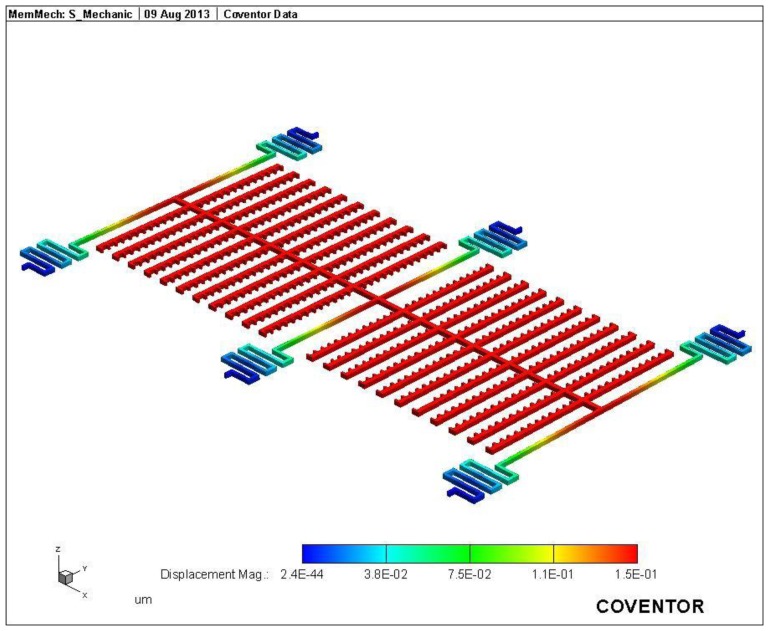
Displacement distribution of the magnetic sensor (unit: μm).

**Figure 4.**
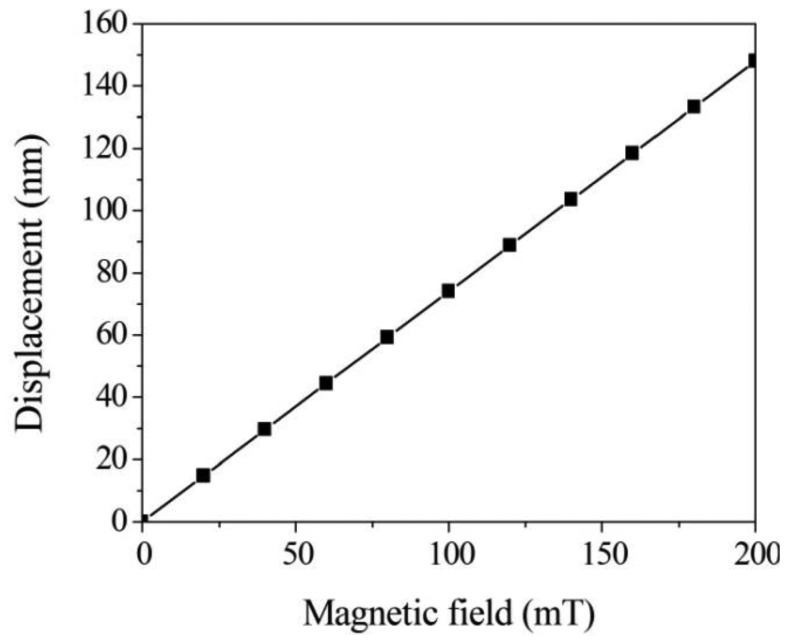
Displacement of interdigitated electrodes in the magnetic sensor.

**Figure 5.**
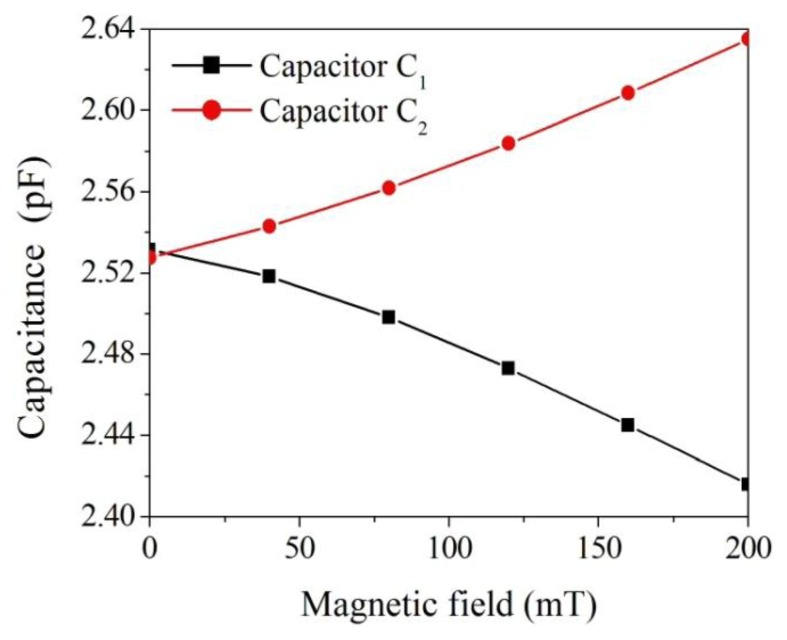
Simulation of capacitance variation in the magnetic sensor.

The FEM software CoventorWare was employed to simulate the displacement and capacitance of interdigitated electrodes in the magnetic sensor. As shown in Figure 1, the model of the magnetic sensor is established. The Manhattan bricks mesh type was used to mesh the sensor model. The parabolic hex element type was adopted, and the amount of elements was 5,540. Materials of the magnetic sensor consist of aluminum, tungsten, polysilicon and silicon dioxide. The Young's moduli of the materials are as follows: aluminum, 77 GPa; tungsten, 400 GPa; polysilicon, 160 GPa; silicon dioxide, 70 GPa. The Poisson's ratios of the materials are as follows: aluminum, 0.3; polysilicon, 0.22; tungsten, 0.28; silicon dioxide, 0.17. Figure 3 shows the displacement distribution of the magnetic sensor. In this simulation, a current of 40 mA and a magnetic field of 200 mT are applied to the magnetic sensor. The results show that the interdigitated electrodes have a maximum displacement of 150 nm. Furthermore, the displacement of interdigitated electrodes is evaluated for different magnetic fields. Figure 4 presents the relation between the displacement of interdigitated electrodes and the magnetic field at 40 mA current. The results show that the displacement of interdigitated electrodes is 75 nm at 100 mT. The capacitances *C_1_* and *C_2_* depend on the displacement of the interdigitated electrodes. CoventorWare also can simulate the variation of the capacitances *C_1_* and *C_2_* in the magnetic sensor under different magnetic fields. Figure 5 shows the simulated results of capacitance variation for the capacitances *C_1_* and *C_2_*. In this investigation, a current of 40 mA is used. The results show that the capacitance *C_1_* increases from 2.53 to 2.63 pF in the magnetic field range of 0–200 mT, and the capacitance *C_2_* decreases from 2.53 to 2.42 pF in the magnetic field range of 0–200 mT.

**Figure 6.**
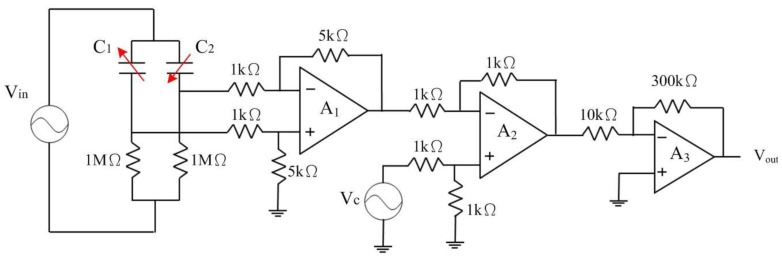
Sensing circuit for the magnetic sensor.

**Figure 7.**
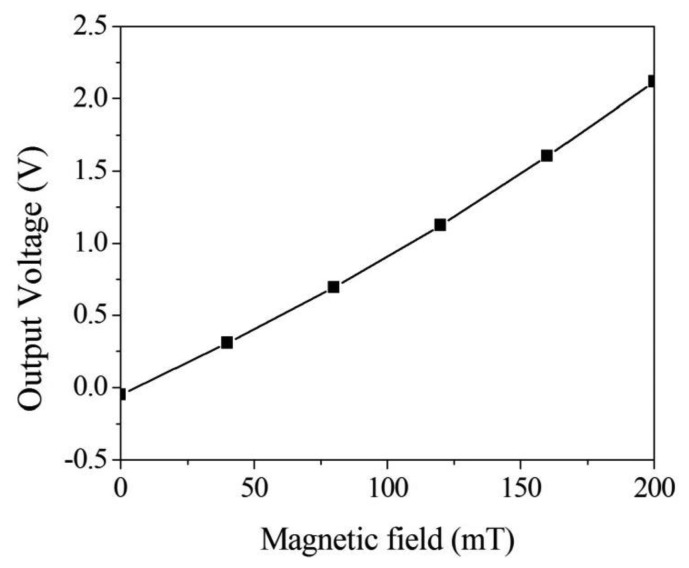
Simulation of output voltage for the sensing circuit.

Figure 6 illustrates the sensing circuit for the magnetic sensor, where *A_1_* represents the differential amplifier, *A_2_* is the differential amplifier, *A_3_* is the inverting amplifier, *C_1_* and *C_2_* are the capacitances in the magnetic sensor [23]. The differential amplifier *A_1_* is utilized to reduce common-mode signals and parasitic capacitances. The differential amplifier *A_2_* is used to offset the signal resulting from the residual stress and thermal expansion of the interdigitated electrodes. The inverting amplifier, *A_3_*, is employed to amplify the output voltage of the differential amplifier, *A_2_*. When the magnetic sensor senses a magnetic field, the capacitances of the magnetic sensor generate a change. The capacitance variation of the magnetic sensor is converted by the sensing circuit into the output voltage. The professional circuitry software HSPICE was used to evaluate the output voltage of the sensing circuit. Figure 7 shows the simulated results of the output voltage for the sensing circuit. In this evaluation, the values in Figure 6 are introduced to the capacitances *C_1_* and *C_2_*. The results show that the magnetic sensor with the sensing circuit has an output voltage of 2.1 V at a magnetic field of 200 mT.

## 3. Fabrication of the Magnetic Sensor

**Figure 8.**
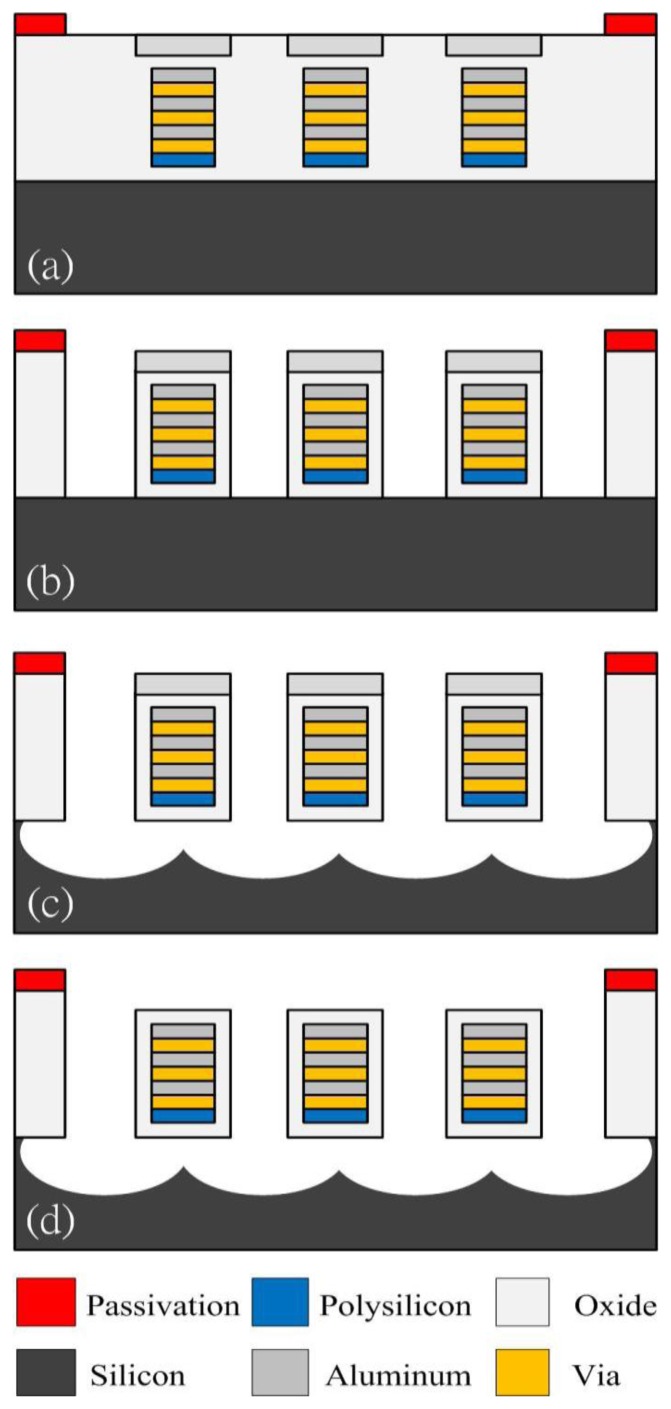
Fabrication flow of the magnetic sensor, (**a**) after the CMOS process; (**b**) removing the oxide layer; (**c**) etching the silicon substrate; (**d**) removing the aluminum hard mask.

The magnetic sensor was fabricated using the commercial 0.35 μm CMOS process of the Taiwan Semiconductor Manufacturing Company (TSMC, Taipei, Taiwan). Figure 8 illustrates the fabrication flow of the magnetic sensor.

**Figure 9.**
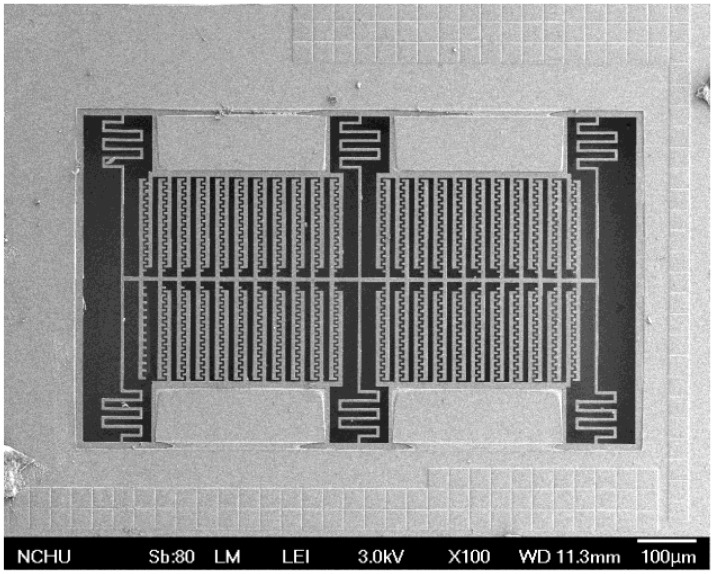
SEM image of the magnetic sensor.

Figure 8a presents the cross-section of the magnetic sensor after completion of the CMOS process. The magnetic sensor needed a post-process [24–26] to release the suspended structure. Figure 8b shows that the sacrificial oxide layer is removed. An anisotropic dry etching of reaction ion etching (RIE) with CHF_3_ (32 sccm), O_2_ (8 sccm), pressure (400 mT) and power (100 W) was used to etch the sacrificial oxide layer [27], and to expose the silicon substrate. Figure 8c displays that the silicon substrate is etched. An isotopic dry etching of RIE with SF_6_ (26 sccm), O_2_ (5 sccm), pressure (100 mT) and power (150 W) was employed to remove the silicon substrate [28,29], and to release the suspended structure. The top layer of the suspended structures was aluminum as a hard mask for protecting the main structure during the anisotropic etching of RIE with CHF_3_/O_2_. The aluminum hard mask must be removed since it increases the residual stress in the structure of the magnetic sensor. Figure 8d shows that the top layer of the suspended structure is removed. An etchant of H_3_PO_4_/HNO_3_/CH_3_COOH/H_2_O (16:1:1:2) at 60 °C was utilized to etch the aluminum hard mask, and to obtain the magnetic structure. As shown in Figure 8d, the magnetic sensor is composed of aluminum, via and oxide layers, in which material of via layer is tungsten. The thickness of each material layer is about 1 μm. Figure 9 depicts a scanning electron microscope (SEM) image of the magnetic sensor after the post-process.

## 4. Results and Discussion

**Figure 10.**
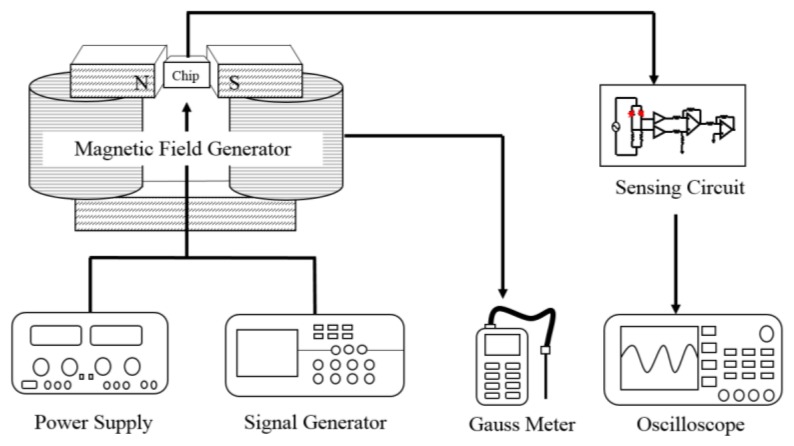
Measurement set-up of the magnetic sensor.

Figure 10 illustrates the experimental set-up of the magnetic sensor. A magnetic field generator, a power supply, a function generator, a gauss meter, and an oscilloscope were used to measure the performance of the magnetic sensor. The power supply was utilized to provide a current to the magnetic field generator. In addition, the power supply was also used to provide a bias voltage to the sensing circuit and a current to the magnetic sensor. The magnetic field generator was employed to produce a magnetic field for testing the magnetic sensor. The gauss meter was adopted to calibrate the magnetic field produced by the magnetic field generator. The oscilloscope was used to record the output voltage of the sensing circuit.

**Figure 11.**
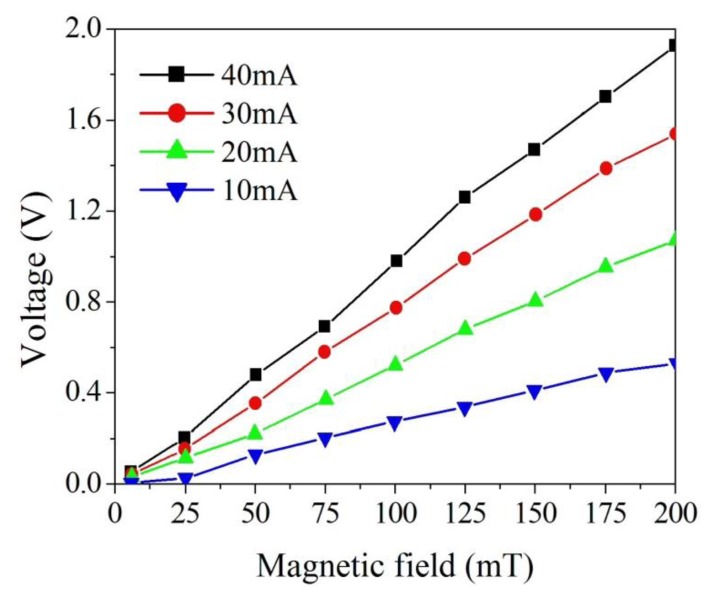
Output voltage of the magnetic sensor.

The magnetic sensor produced a capacitance variation when it sensed a magnetic field. The sensing circuit was employed to convert the capacitance variation of the sensor into the output voltage. As shown in Figure 10, the magnetic sensor chip was set in the magnetic field generator. The function generator supplied a signal to the sensing circuit, and the power supply supplied a current to the sensor chip. At the same time, the power supply provided a current to the magnetic field generator that produced a magnetic field applying to the magnetic sensor chip. The output voltage of the magnetic sensor with the sensing circuit was detected by the oscilloscope. Figure 11 shows the measured results of the output voltage for the magnetic sensor under different supplied currents.

In this measurement, different currents of 10, 20, 30 and 40 mA, respectively, were supplied to the magnetic sensor, and the magnetic field was changed from 5 to 200 mT. The minimum magnetic field detected by the magnetic sensor was 5 mT. The electronic offset voltage of the magnetic sensor for all currents (10, 20, 30 and 40 mA) was 1.5 V. The magnetic sensor burned out if the current was over 50 mA. The results showed that the output voltage of the sensor varied from 0 to 0.45 V at the current of 10 mA when the magnetic field increased from 5 to 200 mT. The output voltage of the sensor changed from 0.01 to 1.03 V at the current of 20 mA in the magnetic field range of 5–200 mT, and its output voltage varied from 0.03 to 1.51 V at 30 mA current in the magnetic field range of 5–200 mT. As shown in Figure 10, the output voltage of the sensor at 40 mA current exceeded that at 20 mA and 30 mA currents, and the output voltage of the sensor increased as the supplied current increased, resulting from the Lorentz force became large as the supplied current enlarged. When the magnetic field changed from 5 to 200 mT, the output voltage of the magnetic sensor at the current of 40 mA increased from 0.05 to 1.94 V. Therefore, the sensitivity of the magnetic sensor was 9.6 V/T. The power consumption of the magnetic sensor was 3.6 mW at the current of 40 mA. As shown in Figure 7, the simulation results showed the output voltage of the magnetic sensor changed from 0.1 to 2.1 V when the magnetic field increased from 5 to 200 mT, so the simulated sensitivity of the sensor was 10.3 V/T. A comparison of the measured and simulated sensitivities for the sensor, the error percentage was 7%, resulting from the fabrication and testing variations.

Beroulle *et al.* [30] proposed a piezoresistive magnetic microsensor fabricated using the CMOS process and a bulk wet etching post-process. The magnetic sensor was actuated by the Lorentz force, and the sensitivity of the sensor was 210 mV/T. Herrera-May *et al.* [31] used MEMS technology to manufacture a resonant magnetic microsensor. The magnetic sensor had a resonant frequency of 22.99 kHz and a sensitivity of 1.94 V/T. Ren *et al.* [32] developed a torsional resonant magnetometer based on MEMS technology. The magnetometer was a capacitive type, and its sensitivity was 400 mV/μT. Domínguez-Nicolás *et al.* [33] presented a resonant magnetic microsensor made using MEMS technology, and the sensor had a sensitivity of 4 V/T. In comparison to Beroulle *et al.* [30], Herrera-May *et al.* [31] and Domínguez-Nicolás *et al.* [33], the sensitivity of this work exceeds that of Beroulle *et al.* [30] and Herrera-May *et al.* [31]. The power consumption of the magnetic sensor proposed by Choi *et al.* [7] was 140 μW, and the magnetic sensor presented by Li *et al.* [8] had a power consumption of 0.58 mW. Comparing to Choi *et al.* [7] and Li *et al.* [8], the power consumption of this work is higher than that of both Choi *et al.* [7] and Li *et al.* [8].

The main advantage of the proposed magnetic sensor is its integration with the sensing circuit that can reduce the parasitic capacitance and the sensor chip area. The post-process of the magnetic sensor is compatible with the commercial CMOS process, so the sensor has potential for mass-production. The main weakness of the magnetic sensor is its high power consumption.

## 5. Conclusions

A magnetic sensor has been manufactured using the 0.35 μm CMOS process and appropriate post-processes. The magnetic sensor contained springs and interdigitated electrodes. The FEM software CoventorWare was employed to simulate the displacement and capacitance of the magnetic sensor. The post-process included an anisotropic dry etching of RIE with CHF_3_/O_2_ to remove the sacrificial oxide layer, followed by an isotropic dry etching of RIE SF_6_/O_2_ to etch the silicon substrate for releasing the suspended structure. Then, an aluminum etchant was used to remove the hard-mask aluminum layer in order to reduce the residual stress of the suspended structure. The post-process was compatible with the commercial CMOS process. The suspended structure in the magnetic sensor, actuated by the Lorentz force, produced a displacement, resulting in a capacitance change of the sensor. The sensing circuit converted the capacitance variation of the sensor into the output voltage. The Lorentz force became large as the current increased, and the output voltage of the senor increased as the current increased. The experimental results showed that the output voltage of the magnetic sensor increased from 0.05 to 1.94 V at the supplied current of 40 mA in a magnetic field range of 5–200 mT. The magnetic sensor had a sensitivity of 9.6 V/T. In the future, we will continue to improve the sensitivity and power consumption of the magnetic sensor and study the sensor's non-linear response, Joule effect, and electronic noise.
